# Cytotoxic Effect of Two Different Concentrations of Sodium Hypochlorite: An In-Vitro Study

**DOI:** 10.7759/cureus.66999

**Published:** 2024-08-16

**Authors:** Divya Mukundan, Ganesh Jeevanandan

**Affiliations:** 1 Pediatric and Preventive Dentistry, Saveetha Dental College and Hospitals, Saveetha Institute of Medical and Technical Sciences, Saveetha University, Chennai, IND; 2 Pediatric and Preventive Dentisty, Saveetha Dental College and Hospitals, Saveetha Institute of Medical and Technical Sciences, Saveetha University, Chennai, IND

**Keywords:** irrigation, pulpectomy, cytotoxicity effect, primary teeth, sodium hypochlorite

## Abstract

Introduction

The endodontic treatment of primary teeth presents considerable complications due to their distinct anatomical properties. In order to achieve a successful endodontic treatment, certain factors must be assessed. These factors include a precise diagnosis, thorough cleaning, and a reliable disinfection protocol. Although sodium hypochlorite (NaOCl) has been effective as an irrigation agent in primary teeth, it is important to recognize that higher concentrations of NaOCl might possibly inflict toxic harm on the periapical environment if they penetrate the tooth’s apical foramina. Since primary teeth are important, pediatric dentists must choose an appropriate NaOCl concentration for root canal irrigation, as higher concentrations can be toxic. Thus, the current investigation examined the cytotoxicity of two different NaOCl concentrations at various volumes.

Methods

To evaluate the cytotoxicity potential, a culture of nauplii (brine shrimp) was prepared and subjected to testing. For the test, 5, 10, 20, and 40 µL of 1% and 3% NaOCl were added to the brine shrimp culture at different concentrations, and saline was used as a control. After a span of 24 hours, the total number of alive nauplii was duly noted.

Results

After 24 hours, nauplii showed no mortality in the control group. For 1% NaOCl, mortality ranged from 10% to 20% across volumes, with no significant differences (p = 0.193). In contrast, 3% NaOCl caused significantly higher mortality: 20% at 5 µL, 30% at 10 and 20 µL, and 60% at 40 µL (p = 0.007). Tukey’s analysis revealed no significant differences for 1% NaOCl (p > 0.05) but significant differences for 3% NaOCl at 40 µL (p < 0.05).

Conclusion

Based on the results of the present study, it was observed that a 1% NaOCl solution exhibited a lower level of toxicity in comparison to a 3% NaOCl solution. These findings highlight the importance of using lower concentrations of NaOCl for endodontic irrigation in pediatric dentistry to reduce the risk of tissue damage and ensure safer outcomes for young patients.

## Introduction

Primary teeth are difficult to treat endodontically due to their unique anatomical characteristics. These include the presence of additional foramina in furcal areas and the distinctive morphology of their root canals [1,2]. Bacteria are considered to be the primary cause of dentinal caries. *Enterococcus faecalis* is the predominant bacterial species commonly found within the root canal. According to studies, *E. faecalis* is resistant to highly alkaline environments [3-5]. To ensure successful endodontic treatment, several key factors must be taken into account. These include making an accurate diagnosis, thoroughly cleaning the canal, using a reliable disinfection protocol, and applying a range of intracanal medicaments. Following these steps, the pulp space should be adequately filled, and the final restoration must be appropriate [6,7]. Based on recent imaging techniques, it has been observed that certain areas of the pulp spaces may not be fully treated through mechanical preparation alone. Therefore, irrigation and instrumentation work together harmoniously to achieve thorough removal of debris and effective disinfection of the root canal [8,9].

Though several irrigation solutions are used in primary teeth, sodium hypochlorite (NaOCl) has always been regarded as the most effective irrigation solution [10]. NaOCl is widely used due to its exceptional antimicrobial properties and ability to effectively dissolve organic matter. It is used in various concentrations, typically ranging from 0.5% to 5.25% [11]. While NaOCl has proven to be a successful irrigation agent for primary teeth, it is important to note that higher concentrations of NaOCl might potentially damage the periapical environment if they penetrate the tooth’s apical foramina [12]. 

Studies carried out in vitro with different NaOCl concentrations revealed that higher concentrations of NaOCl are more toxic than lower concentrations [13,14]. Additionally, studies revealed that preheating NaOCl increased the human pulp tissue’s solvent capacity. Its favorable qualities supported the utilization of a 5.25% NaOCl solution as an irrigant in endodontic procedures, including its ability to dissolve organic tissue, increased antibacterial activity, more alkaline pH, and shorter duration of action [15].

It is essential to exercise caution about the possibility of irrigating solution overflowing through the apical area of primary teeth, as this might cause harm to the underlying permanent tooth. In many case reports, it has been mentioned that higher concentrations of NaOCl are cytotoxic to periapical tissues [16]. An ideal irrigation solution should possess characteristics that are non-toxic, exhibit extensive antimicrobial properties, effectively dissolve necrotic tissue within the pulp, deactivate endotoxins, and prevent the formation of the smear layer [17].

According to reports, lower concentrations of NaOCl showed less cytotoxic effects [17]. Additionally, it is known that higher concentrations of NaOCl solution exhibit cytotoxicity to vital tissues, which occurs as a result of hemolysis, the suppression of neutrophil migration, and damage to endothelial and fibroblast cells [18]. Clinically, this could manifest as pain, ulceration, and delayed wound healing in the periradicular tissues [19]. As a result, an ideal endodontic irrigant should be non-toxic and non-irritating to periodontal tissues. In a case report, it was suggested by Salvadori et al. that a higher concentration of NaOCl possesses the potential to exhibit significant cytotoxicity. The leakage of this material during endodontic treatment may give rise to sequelae, including pain, swelling, bruising, and numbness, associated with chemical burns [20].

Selecting the right NaOCl concentration for root canal irrigation is crucial in pediatric dentistry since higher concentration can have an increased cytotoxic effect and maintaining primary teeth is of paramount importance. Therefore, the objective of this study was to assess and evaluate the extent of cytotoxicity induced by 1% and 3% concentrations of NaOCl at varied volumes.

## Materials and methods

The experimental study was conducted at a research lab in a private dental institution in Chennai in December 2023 and approved by the Institutional Review Board bearing approval no. SRB/SDC/PEDO-2101/23/099.

Preparation of solution

This study used a 3% NaOCl solution from Prime Dental Solutions (Prime Dental Products Pvt Ltd, India), a widely available commercial product. The 1% NaOCl solution was prepared in the research lab by diluting the preceding 3% solution with the following formula:

V1 = 1% × 100 mL divided by 3% = 33 mL.

A calibrated syringe accurately measured 33 mL of a 3% NaOCl solution. It was then transferred to a clean and sterile mixing container. The mixing container was filled with 100 mL of distilled water. Total dilution occurred after gently swirling the liquids. The concentration and production date of the 1% NaOCl solution were clearly labeled. It was kept in a secure area out of direct sunlight and heat. The solution was used for testing within seven days of preparation to maintain its effectiveness. The entire procedure, including preparation and storage, was carried out at a controlled room temperature of 23°C (73.4°F).

Brine shrimp lethality assay

The brine shrimp lethality experiment was used to evaluate the cytotoxicity of NaOCl solutions with concentrations of 1% and 3%. On the first day, a tank was constructed for the hatching of brine shrimp. A quantity of 30 g of salt without iodine was added to 1 L of water that had been purified by distillation, and then this solution was placed into the tank of the experimental apparatus. A suitable aeration system was linked to the tank. Approximately 1 g of Artemia salina eggs were added to the tank and allowed to incubate for 24 hours.

Cytotoxicity assessment

**Figure 1:**
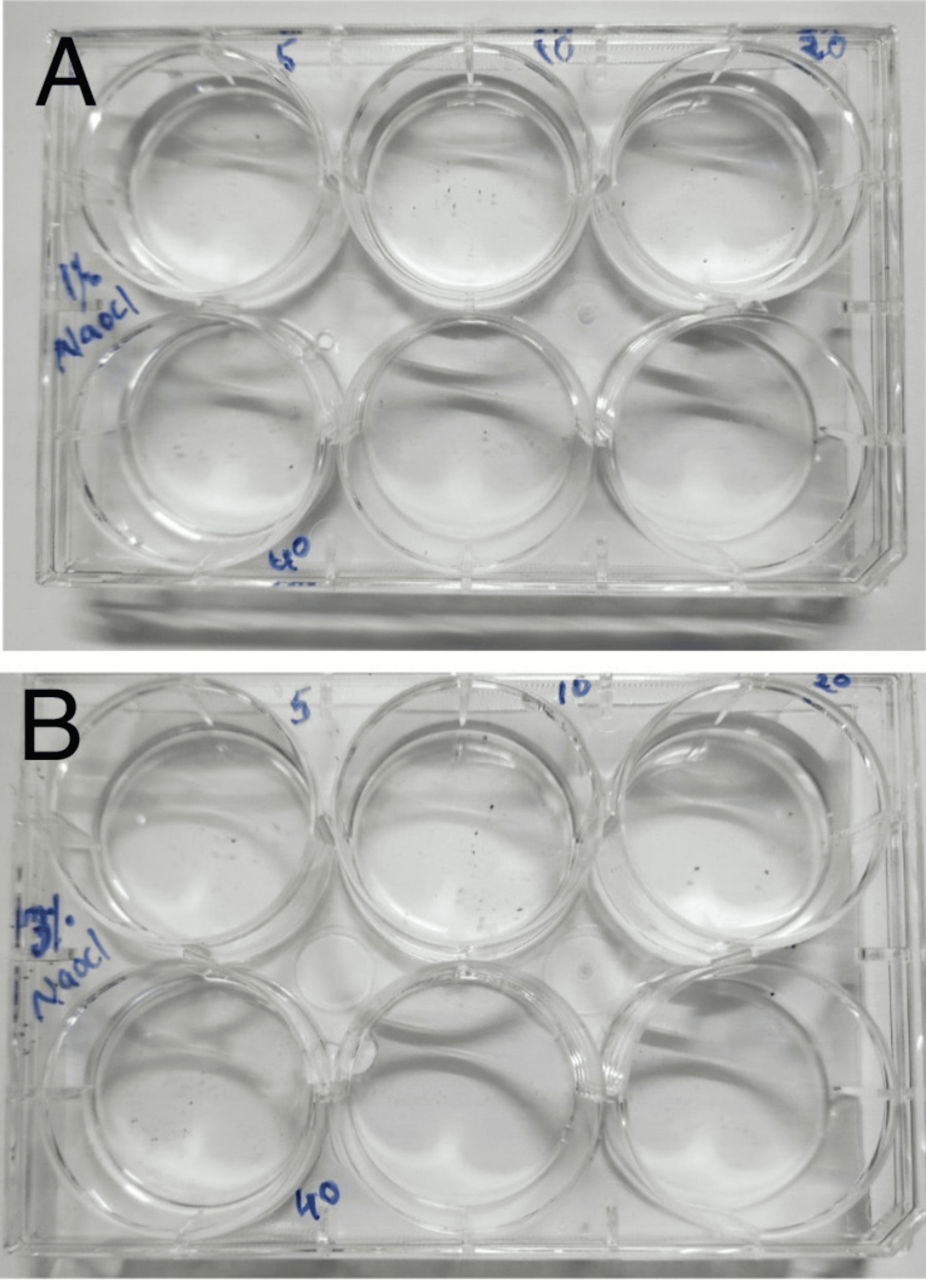
Activity of nauplii in ELISA plates (A) Activity of nauplii in 1% sodium hypochlorite. (B) Activity of nauplii in 3% sodium hypochlorite.

On the second day, precisely 2 g of iodine-free salt was measured and subsequently dissolved in a volume of 200 mL of distilled water. Additionally, saline water was prepared. Each of the six wells of an enzyme-linked immunosorbent assay (ELISA) plate received a total of 10-12 mL of saline water. After an incubation period of 24 hours, a total of 10 nauplii were manually counted and introduced to each well for the experiment by a trained technician. The well was then filled with varying amounts of NaOCl solution, namely 5, 10, 20, and 40 µL, with concentrations of 1% and 3% (Figure 1).

In this study, the control group consists of the saline water containing nauplii by itself. In order to determine the number of live nauplii that were present, the ELISA plates were examined after the incubation period of 24 hours. The number of nauplii in each well was manually counted by a trained technician, and the results were compared to those of the control group. The percentage of live nauplii was calculated using the following formula:

Number of dead nauplii/(number of dead nauplii + number of live nauplii) × 100.

Statistical analysis

Data were analyzed using IBM SPSS Statistics, version 24.0 (IBM Corp., Armonk, NY). A post hoc test was used to assess the inter-group differences individually. Significance was kept at less than 0.05.

## Results

After 24 hours, the live nauplii were evaluated. In the control group, there was no mortality. For the 1% NaOCl treatment, mortality ranged from 10% to 20% across all volumes (5-40 µL), but these results were not statistically significant (p = 0.193). However, the 3% NaOCl treatment showed a significant increase in mortality, with rates of 20% at 5 µL, 30% at 10 and 20 µL, and 60% at 40 µL, which was statistically significant (p = 0.007). These findings suggest that higher concentrations and volumes of NaOCl have a dose-dependent toxic effect on nauplii (Table 1).

**Table 1:** Cytotoxicity activity of different samples

Variable	Volume	No. of live nauplii at baseline	No. of live nauplii after 24 hours	% Mortality	p-value
Control	40 µL	10	10	0%	-
1% Sodium hypochlorite	5 µL	10	9	10%	0.193
	10 µL	10	9	10%	
	20 µL	10	9	10%	
	40 µL	10	8	20%	
3% Sodium hypochlorite	5 µL	10	8	20%	0.007
	10 µL	10	7	30%	
	20 µL	10	7	30%	
	40 µL	10	4	60%	

The cytotoxicity of various concentrations of NaOCl at different volumes was assessed in this study using Tukey’s post hoc analysis. On assessing the 1% NaOCl solution across all three volumes, it was found that no statistically significant differences had been observed, with a p-value of >0.05. This finding signifies that, in the case of 1% concentration, the volumes that were tested yielded comparable results. On the contrary, upon assessing the impact of the concentration of 3% NaOCl, a significant difference was observed at a volume of 40 µl, with a p-value of < 0.05 (Table 2).

**Table 2:** Tukey’s post hoc analysis used to assess the cytotoxicity across different volumes

Dependent variable	(I) Groups according to concentration	(J) Groups according to concentration	Sig
1% Sodium hypochlorite	5 µL	10 µL	0.630
		20 µL	0.630
		40 µL	0.144
	10 µL	5 µL	0.630
		20 µL	1.000
		40 µL	0.630
	20 µL	5 µL	0.630
		10 µL	1.000
		40 µL	0.630
	40 µL	5 µL	0.144
		10 µL	0.630
		20 µL	0.630
3% Sodium hypochlorite	5 µL	10 µL	0.630
		20 µL	0.44
		40 µL	0.005*
	10 µL	5 µL	0.630
		20 µL	0.630
		40 µL	0.026*
	20 µL	5 µL	0.144
		10 µL	0.630
		40 µL	0.004*
	40 µL	5 µL	0.005*
		10 µL	0.026*
		20 µL	0.004*

## Discussion

Over the past few years, there has been a noticeable change in the approach to root canal treatment for primary teeth. Due to the unique characteristics of endodontic infections and the intricate process involved in treating primary teeth, it is recommended that relying only on mechanical instrumentation may not effectively achieve a bacteria-free root canal treatment [21]. Irrigation plays an essential part in the successful completion of endodontic treatment. Throughout the process of instrumentation, the irrigants play a crucial role in aiding the elimination of microorganisms, tissue debris, and dentin remnants from the root canal. This is accomplished by employing a flushing mechanism. Nevertheless, it is important to note that certain irrigating solutions possess cytotoxic properties, which can potentially lead to discomfort and intense pain if they inadvertently penetrate the periapical tissues. The ideal irrigant should possess all or the majority of the favorable attributes while being devoid of any adverse properties [22]. 

NaOCl is currently the preferred irrigant for primary teeth. It is a remarkable broad-spectrum antimicrobial agent that has demonstrated its efficacy against a wide range of oral bacteria, bacteriophages, spores, yeasts, and viruses [13]. Walker and Alfred were the first researchers to consider NaOCl as an irrigant [23], while Salazar et al. evaluated the cytotoxic potential of NaOCl using meristematic root cells [13]. However, it is crucial to be aware that these higher concentrations can also lead to potential harm to the periapical tissues. Particularly when dealing with pediatric patients, endodontic irrigants must be non-toxic to periapical tissues. Case reports have reported the cytotoxic effects of higher concentrations of NaOCl on periapical tissues [16,24]. Taking this into consideration, we undertook the current study to assess the cytotoxicity of NaOCl at two different concentrations.

The rationale for selecting these specific concentrations was to balance efficacy and safety. The 1% concentration was chosen based on previous studies suggesting its lower cytotoxicity while still maintaining sufficient antimicrobial properties [14]. Conversely, the 3% concentration was included to evaluate its safety profile with the lower concentration. The brine shrimp assay was chosen for its simplicity, cost-effectiveness, and rapid results, making it ideal for preliminary cytotoxicity screening. There has been no prior research comparing the cytotoxic effects of various concentrations of NaOCl at different volumes. In the current study, it was confirmed that a concentration of 3% NaOCl had higher cytotoxicity at a volume of 40 µL, with a statistically significant difference that only four live nauplii were found. Conversely, the concentration of 1% NaOCl demonstrated no significant difference. These findings align with the results of Mukundan et al.’s investigation, which concluded that a reduced concentration of NaOCl may be used as an endodontic irrigant because of its minimal cytotoxic effects [14]. Singh et al. found that the proliferation of cultivated fibroblasts was detrimentally impacted by a higher concentration of NaOCl [25]. D’Ercole et al. discovered that 1% NaOCl in combination with non-coherent light-emitting diodes takes five minutes to suppress all *E. faecalis* strains [26]. However, the study by Mensudar et al., 2015 [27] found that a higher concentration of NaOCl is needed for complete smear layer removal.

Utilizing harmful antimicrobial substances in the root canal might potentially disrupt the recovery of periapical tissue. Multiple studies have suggested that the essential components for regeneration involve the cellular expansion, multiplication, and production of fibroblasts’ extracellular matrix [17,25]. Reports have suggested that periapical tissue damage is more likely in cases where the pulp is necrotic or in teeth with root canals that have large apical foramina. This increased risk is due to the higher likelihood of solutions used in the canal passing beyond the apical foramen. However, further research is needed to assess the clinical significance, as factors such as the agent’s concentration, duration of exposure, and exposure surface area significantly influence the outcome. It is important to consider all these factors when choosing the most appropriate agent.

However, the present study has several limitations. The use of a brine shrimp assay, while effective for preliminary cytotoxicity screening, may not fully replicate the complex interactions within human tissues. Additionally, the short 24-hour assessment period may not capture longer-term cytotoxic effects. The study’s scope was also limited to a single species, which could impact the generalizability of the findings to human dental tissues. Future research should explore the cytotoxic effects of NaOCl using more clinically relevant models, extended assessment periods, and a broader range of concentrations and volumes to better inform clinical practice.

## Conclusions

The current study demonstrates that a 1% NaOCl solution exhibits significantly lower cytotoxicity compared to a 3% solution, particularly at higher volumes. It also indicates that lower concentrations of these solutions exhibit reduced toxicity. The findings underscore the importance of selecting the appropriate concentration of NaOCl for endodontic irrigation in pediatric dentistry to minimize potential toxic effects on periapical tissues. Lower concentrations of NaOCl can effectively reduce the risk of tissue damage, ensuring safer outcomes for young patients.
